# Association between low body weight and cytochrome P‐450 enzyme activity in patients with anorexia nervosa

**DOI:** 10.1002/prp2.615

**Published:** 2020-06-11

**Authors:** Pål Sandvik, Stian Lydersen, Solfrid Hegstad, Olav Spigset

**Affiliations:** ^1^ Department of Psychiatry St. Olav University Hospital Trondheim Norway; ^2^ Regional Centre for Child and Youth Mental Health and Child Welfare – Central Norway Trondheim Norway; ^3^ Department of Clinical Pharmacology St. Olav University Hospital Trondheim Norway; ^4^ Department of Clinical and Molecular Medicine Norwegian University of Science and Technology Trondheim Norway

**Keywords:** anorexia nervosa, body weight, cytochrome P‐450, drug treatment

## Abstract

Very little is known to which extent severe underweight could affect cytochrome P‐450 (CYP) enzyme activity. In this study, 24 patients with anorexia nervosa at two occasions ingested single oral doses of five test drugs known to be metabolized by CYP1A2, CYP2C9, CYP2C19, CYP2D6, and CYP3A4, respectively. A mixed model analysis was used to evaluate the effect of changes in body mass index (BMI) on the metabolic activities of these enzymes. The primary end point was the change in drug/metabolite ratio of each of the test drugs per kg/m^2^ change in BMI. With increasing BMI, the metabolic activity of CYP3A4 decreased (change in the CYP3A4 drug/metabolite ratio per unit change in BMI = 0.056; 95% confidence interval [CI] 0.011 to 0.102; *P* = .017). For CYP1A2, increasing BMI increased the metabolic activity with borderline significance (change in the CYP1A2 drug/metabolite ratio per unit change in BMI = –0.107; CI –0.220 to 0.005; *P* = .059). For CYP2C9, CYP2C19, and CYP2D6, no significant changes were seen. The clinical impact of these findings for drug treatment in patients with anorexia nervosa and other severely underweight patients needs to be further studied by examining the pharmacokinetics of specific drugs. This might be particularly relevant for drugs metabolized by CYP1A2 and/or CYP3A4.

**Abbreviations**
BMI: body mass index
CYP: cytochrome P‐450

## 1 INTRODUCTION

About 80% of currently used medicines are metabolized by cytochrome P‐450 (CYP) enzymes.^1^, ^2^ If the activity of these enzymes is altered related to changes in body weight or nutritional state, the plasma concentration of relevant drugs will be affected. The major CYP enzymes involved in drug metabolism include CYP1A2, CYP2C9, CYP2C19, CYP2D6, and CYP3A4.^1^

For CYP1A2, CYP2C9, CYP2C19, and CYP2D6, there are no studies on enzyme activities in anorexia nervosa or otherwise underweight subjects. In contrast, the effects of obesity on CYP enzyme activities have been widely studied (for a review, see 3). The total clearance of drugs metabolized by CYP1A2, CYP2C9, CYP2C19, and CYP2D6 is generally slightly increased in obesity, whereas clearances for drugs that are CYP3A4 substrates are generally reduced by 10%‐35% compared to normal‐weight individuals.^3^, ^4^ The only CYP enzyme for which some data exist in underweight subjects is CYP3A4. A preliminary study found that CYP3A4 enzyme activity was increased in patients with anorexia nervosa compared to a control group with normal weight, and that CYP3A4 activity was negatively correlated with body weight.^5^ Also in healthy volunteers CYP3A4 activity decreases with increasing body weight.^6^ Finally, a negative correlation has been found between body weight and CYP3A4 protein expression in the liver and intestine.^7^

Due to the lack of information on the possible effect of low body weight on most CYP enzymes, the aim of this study was to explore whether changes in body weight in patients with anorexia nervosa affect the metabolic activity of the CYP enzymes CYP1A2, CYP2C9, CYP2C19, CYP2D6, and CYP3A4.

## 2 METHODS

### 2.1 Subjects

Patients with a diagnosis of anorexia nervosa according to the DSM‐5 criteria were included in the study. Key features for a diagnosis of anorexia nervosa include (a) restriction of energy intake, leading to a significantly low body weight; (b) intense fear of gaining weight or persistent behavior that interferes with weight gain; and (c) disturbance in the way the patient experience his or her body weight or shape, or denial of the seriousness of a current low body weight. Exclusion criteria were age below 16 years, pregnancy, lactation, known drug abuse, and unwillingness to give informed consent. The study was approved by the Regional Committee for Medical and Health Ethics in Mid Norway and was conducted according to the Declaration of Helsinki. All patients gave their written informed consent prior to inclusion, and for those under 18 years of age, consent was also given by the patient's legal guardian.

The height of each subject was measured with standard methods, body weight (with light clothes) was weighed on calibrated digital weights, and body mass index (BMI) was calculated accordingly. After inclusion, blood samples were obtained for the analysis of standard biochemical variables, including plasma concentrations of creatinine and hepatic enzymes. There were no significant deviations from the reference intervals. Blood samples were also obtained for genotyping of CYP2C9, CYP2C19, and CYP2D6. Smoking status was evaluated by analyzing the nicotine metabolite cotinine in urine. Patient demographics are summarized in Table 1.

**Table 1:** Demographic characteristics of the 24 patients with a diagnosis of anorexia nervosa included in study

	Mean ± SD	Range
Females/males (n)	23/1	—
Age at inclusion (years)	24.0 ± 6.2	17‐48
Height (cm)	169.4 ± 6.7	160‐187
Body weight at test day 1 (kg)	49.5 ± 6.8	31.3‐62.4
BMI at test day 1 (kg/m^2^)	17.3 ± 2.3	11.8‐20.0
Time interval between tests (days)	109 ± 110	31‐556
Body weight at test day 2 (kg)	54.9 ± 7.2	33.7‐68.2
BMI at test day 2 (kg/m^2^)	19.1 ± 2.1	12.7‐23.6
Percent change in BMI from test day 1 to 2 (%)	11.7 ± 12.0	−14.8 to +39.3

Abbreviations: BMI, body mass index; SD, standard deviation.

### 2.2 Testing of CYP enzyme activities

To evaluate the metabolic activity of the CYP enzymes, the subjects ingested a single oral dose of the following test drugs at two different occasions at least 4 weeks apart: 100 mg of the CYP1A2 substrate caffeine (Koffein, Kragerø tablettproduksjon), 25 mg of the CYP2C9 substrate losartan (Cozaar, MSD), 20 mg of the CYP2C19 substrate omeprazole (Omeprazole Bluefish, Bluefish Pharmaceuticals), 50 mg of the CYP2D6 substrate dextromethorphan (Dexofan, Nycomed Pharma), and 250 mg of the CYP3A4 substrate quinine (Kinin, Kragerø tablettproduksjon). Given concomitantly, these drugs have previously been validated for simultaneous evaluation of the activities these enzymes in the “Karolinska cocktail” (substitution of dextromethorphan with debrisoquine) and the “Inje cocktail” (substitution of quinine with midazolam).^8^, ^9^ The concentration of the drugs as well as of their principal metabolites (paraxanthine, EXP‐3174, 5‐OH‐omeprazole, dextrorphan and 3‐OH‐quinine, respectively) was measured in plasma. Dextromethorphan was ingested 13 hours before blood sampling, caffeine was ingested 4 hours before blood sampling, and losartan and omeprazole were ingested 3 hours before blood sampling.^8^, ^9^ The next day, quinine was ingested separately because it is a powerful CYP2D6 inhibitor, and a blood sample was obtained 16 hours later. None of the other drugs have been found to interact with each other when given concomitantly.^8^

The subjects were asked to abstain from coffee and other products containing caffeine including cola‐like soft drinks, energy drinks, chocolate, or tea, from 36 hours before to 4 hours after intake of the study dose of caffeine.

### 2.3 Analytical methods and calculations

Blood samples (10 ml collected in EDTA tubes) for drug analyses were mixed after sampling and centrifuged for 10 minutes. Plasma was transferred to Eppendorf tubes (Eppendorf, Hamburg, Germany) and stored at −80°C until analysis. Urine samples for the analysis of cotinine and blood samples for CYP genotyping were frozen immediately and stored at −80°C until analysis. The analytical methods for the drugs and their metabolites were based on previously published studies,^10^, ^11^ whereas the method for cotinine was developed at our laboratory (see Data S1). The genotyping methods for CYP2C9, CYP2C19, and CYP2D6 and the variant alleles included are presented as Data S1.

The caffeine/paraxanthine ratio was calculated as a measure of CYP1A2 activity, the losartan/EXP‐3174 ratio was calculated as a measure of CYP2C9 activity, the omeprazole/5‐OH‐omeprazole ratio was calculated as a measure of CYP2C19 activity, the dextromethorphan/dextrorphan ratio was calculated as a measure of CYP2D6 activity and the quinine/3‐hydroxyquinine ratio was calculated as a measure of CYP3A4 activity. Changes in the drug/metabolite ratios were calculated to evaluate any influence on the activity of the related CYP enzyme. As the distributions of the metabolic ratios were found to be heavily right‐skewed, the natural logarithm (ln) of the metabolic ratios was employed as the outcome variable in the statistical model to achieve near normality.

The creatinine‐adjusted cotinine concentration (ie, µg cotinine per mg creatinine) in urine was used to estimate the degree of smoking.^12^

Patients who turned out to be genotypically poor metabolizers for CYP2C9, CYP2C19, or CYP2D6 (Table S1) were excluded from the analysis of that enzyme since they did not produce any active enzyme and could therefore not be subject to any weight‐related alterations in the metabolic capacity. This was the case for to two patients for CYP2C9 and two patients for CYP2D6.

### 2.4 Statistics

The number of patients included in the study was based on the possibility to detect a 30% change in drug clearance between the two test occasions. Such a difference was considered to correspond to a difference in drug/metabolite ratio (logarithmic values) of about 0.8, based on combined data for the effect of oral contraceptives on the CYP2C19 drug/metabolite ratio for the probe drug omeprazole as well as on the clearance of omeprazole.^13^, ^14^ The sample size calculation was carried out for a repeated measures study design with two time points, with a standard deviation of 0.7 for single observations^15^ and a correlation between the two observations of 0.5, using the software NCSS PASS. To obtain a power of 0.80 with a significance level of 0.05, a total of 20 individuals were found to be required. To allow for exclusion of patients being poor metabolizers via some of the enzymes, 24 patients were included in the study.

Since data from two time points were included from each patient, a mixed model analysis was applied. The model assumed that the metabolic ratios of each individual patient possess a random intercept (ie, an individual “offset”) in addition to being affected by the patient's BMI at the time of sampling. Thus, we used natural logarithm of the metabolic ratio as dependent variable, patient as random factor, time (after versus before) as fixed factor, and BMI as covariate. The model assumes that the changes in metabolic ratios on the logarithmic scale are linear across the BMI range studied. As some patients used drugs inhibiting the activity of CYP2D6 concomitantly, use of CYP2D6 inhibitors was included in the model for this enzyme. Weak inhibitors (citalopram, escitalopram) were given a score of 1, whereas strong inhibitors (fluoxetine, paroxetine) were given a score of 2. Normality of residuals was confirmed by visual inspection of QQ plots. Results are presented as means with 95% confidence intervals. To compare correlations between two variables, Pearson's correlation test was used. Two‐sided *P* values <.05 were considered statistically significant. The software used was SPSS 25.

## 3 RESULTS

In total, 24 patients were included in the study. Mean BMI was 17.3 kg/m^2^ the first test day and 19.1 kg/m^2^ the second test day, and the intraindividual changes in body weight between the two test days varied from −14.8% to +39.3% (Table 1). The mean time interval between the two test occasions was 109 days. BMI values at the first and second test day for each patient as well as the number of days between the two test occasions at the individual level are shown in Figure S1. Crude data for the metabolic ratios at the two test days are presented in Table S2.

**FIGURE 1:**
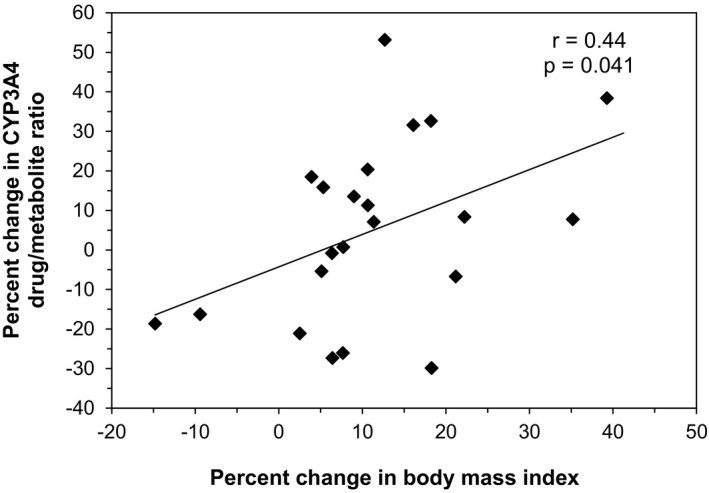
Correlation between relative change in body mass index and relative change in the CYP3A4 drug/metabolite ratio (logarithmic values) in the 24 patients with anorexia nervosa included in the study. The r and p values represent Pearson's correlation

Results from the mixed model analysis are presented in Table 2. With increasing BMI, the estimate for the CYP3A4 drug/metabolite ratio was significantly positive (*P* = .017), that is the metabolic activity of CYP3A4 decreased with increasing BMI. The correlation between the change in BMI and the change in CYP3A4 drug/metabolite ratio at an individual level is depicted in Figure 1, and was also statistically significant (*r* = .44, *P* = .041). For CYP1A2, increasing BMI gave a negative estimate for the drug/metabolite ratio of borderline significance (*P* = .059), that is a possible increase in metabolic activity. For the other enzymes, no significant relationships between BMI and metabolic ratios were found (Table 2). Co‐treatment with CYP2D6 inhibitors was found to significantly increase the drug/metabolite ratio, that is a decrease in the CYP2D6 metabolic activity (*P* = .004) (Table 2).

**Table 2:** Linear mixed effect regression with the natural logarithm of the parent substance/metabolite ratio for the test drug metabolized by the enzyme in question as dependent variable, and body mass index (BMI; kg/m^2^) and time point (after vs. before) as covariates, and patient as random effect, for the 24 patients included in the study (22 patients were included for CYP2C9 and CYP2D6 because two patients who were poor metabolizers for each of these enzymes were excluded)

Enzyme	Variable	Estimate	95% confidence interval	*P* value
CYP1A2	BMI	−0.107	−0.220 to 0.005	0.059
	Smoking^a^	−0.278	−0.590 to 0.035	0.080
CYP2C9	BMI	0.040	−0.070 to 0.150	0.466
CYP2C19	BMI	0.012	−0.092 to 0.116	0.822
CYP2D6	BMI	0.052	−0.103 to 0.207	0.499
	Inhibitor^b^	0.715	0.246 to 1.184	0.004
CYP3A4	BMI	0.056	0.011 to 0.102	0.017

Note

Estimated regression coefficients are shown for body mass index (BMI), for CYP1A2 also for smoking and for CYP2D6 also for concomitant treatment with enzyme inhibitors.

^a^ Measured as the creatinine‐adjusted cotinine concentration (ie, µg cotinine per mg creatinine) in urine, which was used as a continuous variable to estimate the degree of smoking.

^b^ Effect of concomitant treatment with a drug acting as a CYP2D6 inhibitor. Weak inhibitors (citalopram, escitalopram) were given a score of 1, whereas strong inhibitors (fluoxetine, paroxetine) were given a score of 2. For further details, see text.

The estimate for smoking (measured by the cotinine/creatinine ration in urine) related to the drug/metabolite ratio for CYP1A2 was negative, implying that the metabolic activity via CYP1A2 was increased in smokers, although the confidence interval was wide and the effect was not statistically significant (*P* = .080). However, as urine was not available from five subjects at test day 1 and from six subjects at test day 2, these subjects had to be excluded from the CYP1A2 and smoking regression. Due to the obvious risk of type II errors by excluding these subjects from the analysis, we also checked the effect of BMI on CYP1A2 without including smoking status in the model (n = 24). We then found a statistically significant relationship (estimate −0.123, 95% confidence interval −0.219 to −0.027, *P* = .014).

## 4 DISCUSSION

The principal finding in this study was that the metabolic activity of CYP3A4 decreased with increasing BMI. In addition, a possible, but nonsignificant, positive correlation between BMI and the metabolic activity of CYP1A2 was found.

The effects of low body weight on the activity of most of these enzymes have not been studied before, but it would be reasonable to believe that as the body saves energy in severe underweight, the metabolic activity of CYP enzymes could also be lower, and gradually increase as the body weight normalizes. In fact, such an effect was possibly seen for CYP1A2, only. It should be noted that the statistically significant effect of BMI on CYP1A2 activity disappeared when smoking was included in the model, and that smoking by itself was not found to significantly affect CYP1A2 metabolic rate although smoking is a well‐documented inducer of this enzyme.^16^ We consider the lack of statistical significance for smoking and possibly also for CYP1A2 to be a type II error, as urine was missing for several subjects at both test days and these subjects had to be excluded from the combined regression model. The risk of a type II error is further substantiated by the fact that the BMI estimate for CYP1A2 was principally the same irrespective of whether smoking was included in the analysis.

For CYP3A4, a seemingly paradoxical decreased CYP3A4 metabolic activity was observed with increasing body weight. This effect is principally the same as in another study in patients with anorexia nervosa, measuring the relationship between the endogenously produced CYP3A4 substrate cholesterol and its metabolite 4β‐hydroxycholesterol.^5^ In that study the CYP3A4 activity was also higher in underweight patients with anorexia nervosa than in a group of healthy, normal‐weight controls.^5^ In other studies, obese patients have had even lower CYP3A4 activities than normal‐weight controls.^3^, ^4^, ^17^ Taken together, these data suggest that there might be a continuous inverse relationship between body weight and CYP3A4 activity, extending all the way from severely underweight to obese subjects.

It is complicated to “translate” changes in drug/metabolite ratios into changes in concentrations for drugs metabolized by the enzyme. However, some attempts can be made based on the observed effect of weak CYP2D6 inhibitors on the change in drug/metabolite ratio for this enzyme of 0.715. Such an effect would be expected to be equal to, for example, the effect of escitalopram on the CYP2D6 substrate metoprolol, causing an 89% increase in the metoprolol concentration.^18^ Given that linearity exists and that CYP3A4 ratios can be directly compared to CYP2D6 ratios, an increase in BMI of 13 kg/m^2^ would give an increase in the CYP3A4 drug/metabolite ratio of 0.056 × 13 = 0.728. This is close to 0.715, and given that these prerequisites are correct, thereby corresponding to roughly a doubling of the concentration of a sensitive CYP3A4 substrate. An increase in BMI of 13 kg/m^2^ is considerable, for example from a BMI of 12 kg/m^2^ to a BMI of 25 kg/m^2^, but again, the estimate is extremely uncertain, as also illustrated by the wide confidence interval of 0.011 to 0.102 for the BMI effect of CYP3A4. There was also a relatively weak correlation between BMI and CYP3A4 enzyme activity, with an r value of 0.44. Nevertheless, this is relatively close to the value of 0.56 found in the previous study using the 4β‐hydroxycholesterol/cholesterol ratio to estimate CYP3A4 activity.^5^

As can be seen from Figure 1, changes in CYP3A4 activity at an individual level are relatively unpredictable. This is not surprising given the many other factors that affect CYP3A4 activity. As weight changes in addition take place relatively slowly, standardized dose changes based on alterations in body weight are not meaningful. Our study nevertheless represents a signal that dose changes might be necessary during conditions affecting body weight also in the underweight range, and that the direction of dose change may differ depending on the specific CYP enzyme involved.

The underlying mechanisms of the observed increased CYP3A4 metabolism and possibly decreased CYP1A2 metabolism in underweight subjects are unclear and should be subject to future research. As 23 of 24 patients in this study were females, one putative explanation could be decreased estrogen levels related to low body weight. During pregnancy, when estrogen and progesterone levels are increased, the activity of CYP1A2 decreases, whereas the activity of CYP3A4 increases.^19^ In addition, the activities of particularly CYP2D6, but also CYP2C9 are increased.^19^ However, it seems unlikely that increased estrogen levels should have the opposite effects on CYP1A2 and CYP3A4 activities in recovering underweight subjects and in pregnancy.

Another possible mechanism could be the influence of pro‐inflammatory cytokines such as tumor necrosis factor alpha (TNF‐α) and interleukin 6 (IL‐6), which have been reported to decrease the activities of most CYP enzymes (for a review, see 20). Patients with anorexia nervosa have elevated concentrations of these cytokines,^21^, ^22^ and if the increase in cytokine concentrations is sufficiently high to affect CYP enzyme activities, it could explain the possibly lower CYP1A2 activity, but not the increased CYP3A4 activity. Of note, the levels of these cytokines are also increased in obesity, further complicating the picture. Thus, the relations between body weight, sex hormones, cytokine levels, and CYP enzyme activities are obviously very complex.

This study has some limitations that should be acknowledged. As already discussed, there is no simple way of “translating” data on metabolic ratios to the impact on metabolism of drugs via the enzyme in question. To elucidate this aspect further, studies in patients using specific drugs are required. Another limitation is the low number of subjects included. Replication in larger materials is therefore wanted. It is also a weakness that a control group with normal‐weight subjects was not included. Moreover, many patients in this study had a BMI above the lower limit in the normal range of 18.5 kg/m^2^, particularly at test day 2, when the mean BMI value was 19.1 kg/m^2^. The time interval between the test days was not standardized and varied considerably between subjects. However, including this interval as a variable in the regression model gave principally the same results, demonstrating that this variation did not impact our findings. Although the intake of caffeine was strictly regulated prior to the test days, there were no other dietary restrictions. Thus, in theory, intake of, for example, grapefruit juice and certain foods including fruits and cruciferous vegetables could have affected the results. However, we consider such a possibility being unlikely. Grapefruit juice is not readily available in Norway, and the foods in question influence CYP activities to a small extent only, particularly in the amounts that patients with anorexia nervosa would be expected to ingest. Finally, it should be emphasized that as this study was performed in patients with anorexia nervosa, we do not know whether the results are valid for other patient groups with low body weight and/or malnutrition.

In conclusion, in patients with anorexia nervosa, the metabolic activity of CYP3A4 decreased, whereas the metabolic activity of CYP1A2 might increase, with increasing BMI. The clinical impact of these findings needs to be further studied by examining the pharmacokinetics of relevant drugs related to body weight and changes in body weight in the underweight range.

## DISCLOSURE

There are no competing interests to declare.

## AUTHOR CONTRIBUTION

PS and OS designed the study; PS recruited patients to the study; PS, SH, SL, and OS did the data analysis and interpretation; OS wrote the manuscript with input from PS, SH, and SL. All the authors have approved the final version of the manuscript.

## Supporting information

Data S1‐Table S1‐S2‐Figure S1

## Data Availability

Data available on request from the authors.
