# Supplementary material for: Association between low body weight and cytochrome P‐450 enzyme activity in patients with anorexia nervosa
Source: Pharmacol Res Perspect. 2020 Jun 11;8(3):e00615. doi: 10.1002/prp2.615 (PMC7290083; doi:10.1002/prp2.615)
Supplement: Supplementary file 1 — Data S1‐Table S1‐S2‐Figure S1 [file PRP2-8-e00615-s001.docx]

Supporting Information

**Association between low body weight and cytochrome P-450 enzyme activity in patients with anorexia nervosa**

**Analysis of test drugs and metabolites**

For the analyses of caffeine, losartan, omeprazole, dextromethorphan and their metabolites, 500 µL was mixed with 200 µL protein precipitation solution containing the internal standard propranolol. Samples were centrifuged and the supernatant were transferred to new tubes and evaporated to dryness. Thereafter, 100 µL of 50% methanol were added to the dry residue and the sample was vortexed and injected onto the HPLC-MS/MS system.

Separation of analytes was performed with a Waters Xbridge C18 column (21 × 100mm, 3.5 µm; Waters, Palo Alto, CA, USA) using gradient run with two mobile phases: water with 0.1% formic acid and acetonitrile with 0.1% formic acid, respectively. Analysis was performed in a HPLC-MS/MS system composed of a Shimadzu LC20AD LC system and an ABSciex tandem- quadrupole mass spectrometer (Shimadzu Scientific Instruments, Columbia, SC, USA). The mass spectrometer was operating in positive mode with electrospray ionization and multiple reaction monitoring. Transitions used and precision data are found in the original publication.

For the analyses of quinine and its metabolite, 100 μL plasma were mixed with 200 µL protein precipitation solution containing the internal standards quinine-d_3_ and 3-OH-quinine-vinyl-d_3_. After vortexing and centrifugation the extract was injected onto the UPLC-MS/MS system.

Separation of the analytes was performed with an Acquity UPLC BEH C18-column (2.1 × 50 mm 1.7 μm; Waters), using gradient run with two mobile phases: 11 mM ammonium formate and 0.1% formic acid in acetonitrile, respectively. The analytes were detected using a Micromass Quattro Premier XE mass spectrometer (Waters) operating in positive mode with electrospray ionization and selected reaction monitoring. Transitions used were *m/z* 325 > 160 for quinine and *m/z* 341 > 160 for 3-OH-quinine. More data about the method is found in the original publication.

*References:*

Thu OKF, Spigset O, Nilsen OG, Hellum B. Effect of commercial Rhodiola rosea on CYP enzyme activity in humans. Eur J Clin Pharmacol 2016; 72: 295-300.

Björkhem-Bergman L, Bäckström T, Nylén H, Rönquist-Nii Y, Bredberg E, Andersson TB, et al. Quinine compared to 4β-hydroxycholesterol and midazolam as markers for CYP3A induction by rifampicin. Drug Metab Pharmacokinet 2014; 69: 352-5.

**Analysis of cotinine and creatinine in urine**

Urinary concentrations of cotinine were determined by a UHPLC-MS/MS method. In brief, pre-treatment and extraction of samples were performed using the pipetting robot platform Tecan Freedom Evo 100 and 150 with MCA96 and TeVacs (Tecan Männedorf, Switzerland). To 0.5 mL of urine, 0.5 mL of 50 mM ammonium acetate (pH 6.0) and 25 µL of the internal standard cotinine-d_3_ (10 ng/mL) were added. The samples were extracted on an Oasis MCX 96 well plate 60 µm/30 mg (Waters, Milford, MA, USA) pre-conditioned with methanol (1 mL) and water (1 mL). The column was washed with water (1 mL), 0.1 M hydrochloric acid (0.5 mL) and methanol (1 mL) and the analytes were eluted with 0.6 mL methanol/ammonium hydroxide (95:5 v/v), evaporated to dryness and dissolved in 200 µL water. Samples above the highest calibration level were diluted in blank urine prior to sample preparation.

# Separation was performed on an Acquity UPLC I-Class FTN system (Waters, Milford, MA, USA) at 50 ºC with an HSST3 column (2.1 × 100 mm, 1.8 µm) (Waters, Wexford, Ireland). The mobile phase consisted of 0.1 % formic acid in water (A) and 100 % methanol (B). Gradient profile (% B) was: 2-25 % in 0.0-0.5 min, 25-80 % B in 0.5-1.9 min, 80-98 % in 1.9-1.96 min, 98 % in 1.96-2.4 min, 98-2 % in 2.4-2.41 min and 2 % in 2.41-2.55 min. The injection volume was 1 µL. MS detection was performed on a Waters Xevo TQ-S tandem-quadrupole MS, using positive electrospray ionization. The *m/z* transitions 177.1 > 98.1 and 177.1 > 80.1 were monitored for cotinine, whereas the *m/z* 180.1 > 101.1 transition was monitored for cotinine-d_3_.

The calibrated range was 0.5 to 500 ng/mL, and the limit of quantification (LOQ) was determined at the lowest calibrator sample. Linearity was shown throughout the calibrated interval (R^2^ > 0.999). The between-assay relative standard deviations were varied from 1.1 % to 4.2 % at concentrations of 0.8, 50.2 and 254 ng/mL.

Creatinine was analyzed photometrically after complex formation with picric acid in an alkaline solution by a routine method (Jaffé’s method) on a Cobas Integra 400+ multianalyzer (Roche Diagnostics, Rotkreutz, Switzerland). The limit of quantitation (LOQ) was 0.23 mg/mL with a relative standard deviation of 3.0 %. The creatinine-adjusted cotinine concentration in urine (i.e. µg cotinine per mg creatinine excreted) was used as a marker for the degree of smoking.

**CYP genotyping**

From EDTA-treated whole blood samples, genomic DNA was extracted using a Qiagen Blood and Cell Culture DNA kit (Qiagen, Hilden, Germany), according to the manufacturer’s guidelines. The CYP2D6*3, *4, *6, *7, *8, *9, *10 and *41 alleles were determined by allele-specific polymerase chain reaction (PCR). In addition, CYP2D6 was analyzed for the gene deletion (CYP2D6*5) as well as for the duplication/multiduplication of the gene. The CYP2C9*2 and *3 alleles, and the CYP2C19*2, *3 and*17 alleles were also identified by PCR. Alleles where none of these mutations were found were classified as *1.

Carriers of two inactivating variants, i.e. any combinations of *3, *4, *5, *6, *7, and/or *8 for CYP2D6, *2 and/or *3 for CYP2C9, or *2 and/or *3 for CYP2C19, were defined as poor metabolizers for that enzyme. Carriers of the CYP2D6 duplication/multiduplication and carriers homozygous for the *17 variant for CYP2C19 were classified as ultrarapid metabolizers.

**Supporting Table 1**

Genotype distribution for CYP2D6, CYP2C19 and CYP2C9 for the 24 patients with anorexia nervosa included in the study.

| **CYP2D6** | | **CYP2C19** | | **CYP2C9** | |
| --- | --- | --- | --- | --- | --- |
| Allele combination | Number | Allele combination | Number | Allele combination | Number |
| *1/*1 | 9 | *1/*1 | 14 | *1/*1 | 17 |
| *1/*4 | 7 | *1/*2 | 3 | *1/*2 | 3 |
| *1/*5 | 1 | *1/*17 | 6 | *1/*3 | 2 |
| *1/*9 | 1 | *17/*17^§^ | 1 | *3/*3^¶^ | 2 |
| *1/*41 | 2 |  |  |  |  |
| *3/*3^†^ | 2 |  |  |  |  |
| *41/*41 | 1 |  |  |  |  |
| Duplication/ multiduplication^‡^ | 1 |  |  |  |  |

^†^ Defined as poor metabolizers for CYP2D6 and excluded from the CYP2D6 analysis

^‡^ Defined as ultrarapid metabolizers for CYP2CD6

^§^ Defined as ultrarapid metabolizers for CYP219

^¶^ Defined as poor metabolizers for CYP2C9 and excluded from the CYP2C9 analysis

**Supporting Table 2**

Metabolic ratios of cytochrome P450 (CYP) enzymes at two occasions in 24 patients with anorexia nervosa. Data are presented as medians with 25-75 percentiles. Interval between test days was 31-556 days (mean 109 days). Mean body mass index (± standard deviation) was 17.3 ± 2.3 kg/m^2^ at test day 1 and 19.1 ± 2.1 kg/m^2^ at test day 2.

|  | **Test day 1** | | **Test day 2** | |
| --- | --- | --- | --- | --- |
|  | Median  (25-75 percentile) | Mean ± SD | Median  (25-75 percentile) | Mean ± SD |
| CYP1A2  (caffeine/paraxanthine  ratio) | 9.8  (3.4-32.0) | 17.8 ±16.4 | 7.7  (2.9-22.6) | 14.5 ±16.5 |
| CYP2C9  (losartan/EXP-3174  ratio) | 0.42  (0.28-0.77) | 1.14 ± 2.64 | 0.68  (0.46-0.94) | 1.48 ± 3.02 |
| CYP2C19  (omeprazole/5-OH-omeprazole ratio) | 0.14  (0.07-0.23) | 0.17 ± 0.12 | 0.10  (0.06-0.13) | 0.18 ± 0.24 |
| CYP2D6  (dextromethorphan/dextrorphan ratio) | 1.2  (0.6-2.9) | 2.8 ± 3.8 | 2.1  (1.1-5.6) | 4.6 ±5.7 |
| CYP3A4  (quinine/3-OH-quinine  ratio) | 4.3  (3.2-5.6) | 4.9 ± 2.2 | 4.3  (3.4-6.0) | 5.6 ± 4.0 |

**Supporting Figure 1**


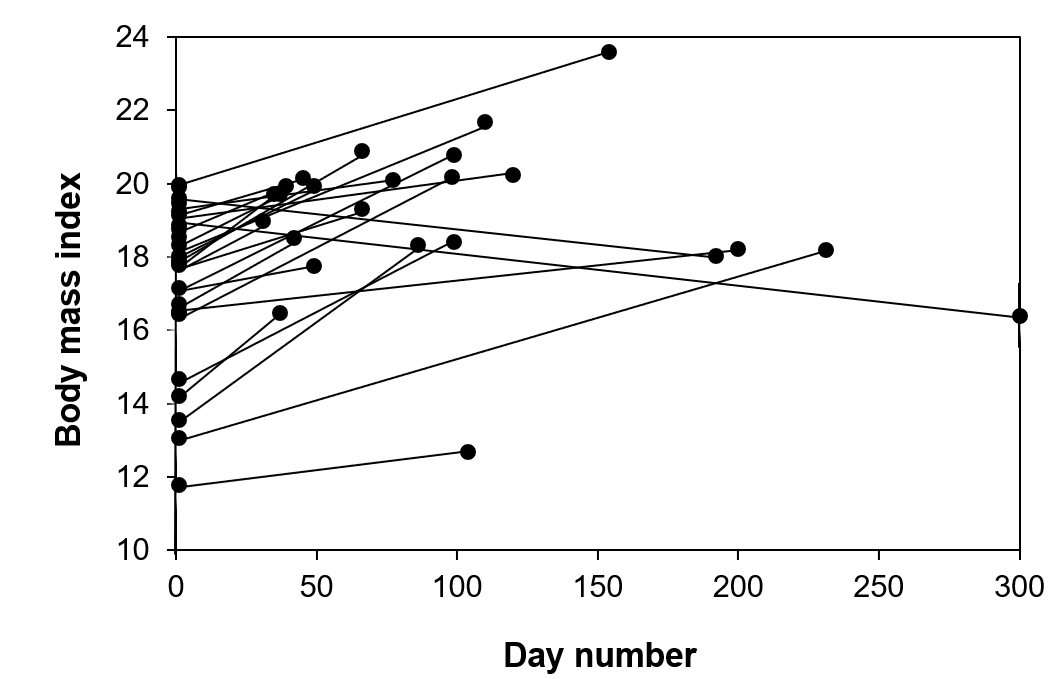


Plot showing the individual changes in body mass index (in kg/m^2^) between the first and the second day of testing for all the 24 patients with anorexia nervosa included in the study. The number of days between the two test days, where day 0 represents the first day of testing, is also displayed. For one patient, the interval between the test days was 556 days. For clarity, the second test day for this patient is shown as day 300.
